# Tetralogy of Fallot or Pulmonary Atresia with Ventricular Septal Defect after the Age of 40 Years: A Single Center Study

**DOI:** 10.3390/jcm9051533

**Published:** 2020-05-19

**Authors:** Julia Hock, Laurent Schwall, Claudia Pujol, Alfred Hager, Renate Oberhoffer, Peter Ewert, Oktay Tutarel

**Affiliations:** 1 Department of Congenital Heart Disease and Paediatric Cardiology, German Heart Centre Munich, Technical University of Munich, 80636 Munich, Germany; julia.hock@tum.de (J.H.); laurentschwall@gmail.com (L.S.); claupujol@gmail.com (C.P.); hager@dhm.mhn.de (A.H.); ewert@dhm.mhn.de (P.E.); 2 Institute of Preventive Paediatrics, Department of Sport and Health Sciences, Technical University of Munich, 80992 Munich, Germany; renate.oberhoffer@tum.de; 3 DZHK (German Centre for Cardiovascular Research), Partner Site Munich Heart Alliance, 80992 Munich, Germany

**Keywords:** outcome, adult congenital heart disease, Fallot

## Abstract

*Background:* The population of adults with tetralogy of Fallot (TOF) or pulmonary atresia with ventricular septal defect (PA/VSD) is growing and aging. Data regarding older patients are scarce. Prognostic outcome parameters in adults with TOF or PA/VSD ≥ 40 years were studied. *Methods*: This was a retrospective study of patients ≥ 40 years of age during the study period (January 2005–March 2018). Major adverse cardiac events (MACE) were a combined primary endpoint including death from any cause, prevented sudden cardiac death, pacemaker implantation, arrhythmia, and new-onset heart failure. Additionally, MACE II (secondary endpoint) was a combination of death from any cause and prevented sudden cardiac death. *Results*: 184 (58.7% female, mean age 45.3 ± 7.2 years) patients were included (159 (86.4%) TOF and 25 (13.6%) PA/VSD). During a median follow-up of 3.1 years (IQR: 0.6–6.5), MACE occurred in 35 and MACE II in 13 patients. On multivariable analysis, New York Heart Association class [HR: 2.1, 95% CI: 1.2–3.6, *p* = 0.009] emerged as an independent predictor for MACE, and age at corrective surgery [HR: 13.2, 95% CI: 1.6–107.1, *p* = 0.016] for MACE II. *Conclusions*: Adults with TOF or PA/VSD ≥ 40 years are burdened with significant morbidity and mortality. New York Heart Association class and age at corrective surgery were independent predictors of outcome.

## 1. Introduction

Due the advances in the treatment of congenital heart disease (CHD), more children survive to adulthood, leading to a new group of patients: adults with congenital heart disease (ACHD). The number of ACHD patients is increasing and patients are reaching older age, leading to an elderly ACHD patient group. Indeed, there is a growing number of ACHD patients over the age of 60 years with high mortality rates and higher utilization of healthcare resources compared with younger patients [1,2]. Acquired morbidities, such as coronary artery disease, seem to be key determinants of outcome in this older population in conjunction with the underlying CHD. Tetralogy of Fallot (TOF) was one of the first CHD repaired by surgery beginning in the 1950s [3], leading to a better life expectancy. Despite advances in treatment during the last six decades, TOF patients continue to be afflicted with increased mortality in comparison to the general population as they grow older [4]. However, while the course of TOF in younger patients has been extensively studied, data regarding patients over the age of 40 years are scarce. Considering the increasing numbers of patients that reach this age threshold and the increased mortality in comparison with the general population, the clinical course of a contemporary cohort of TOF patients over the age of 40 years is of interest. Furthermore, identifying key risk factors for an adverse outcome is of importance.

Therefore, the aim of this study is to examine the clinical course of patients with tetralogy of Fallot or pulmonary atresia with ventricular septal defect (PA/VSD) over the age of 40.

## 2. Materials and Methods

### 2.1. Study Population

This was a retrospective cohort study, including all patients with a diagnosis of TOF or PA/VSD under follow-up at the German Heart Centre Munich who were ≥40 years of age at any point between January 2005 and March 2018. The time-point of inclusion was either the date of the 40th birthday or, if the patient was already 40 years old in the year 2005, the first visit after the 1st of January 2005.

The primary endpoint was the occurrence of a major adverse cardiovascular event (MACE), defined as the occurrence of either death from any cause or prevented sudden cardiac death (SCD) or implantation of a pacemaker or new-onset heart failure or new arrhythmias. A secondary endpoint (MACE II) was defined as either death from any cause or prevented SCD. For these analyses patients with palliation were excluded. Arrhythmias encompassed any type of supraventricular or ventricular arrhythmia requiring therapy. Prevented sudden cardiac death included patients with sustained ventricular arrhythmias (defined as a duration of >30 s or requiring cardioversion) and patients with an appropriate shock by an implantable cardioverter-defibrillator. New-onset heart failure was defined as an initiation of heart failure medication (e.g., loop-diuretics) previously not prescribed. Age at correction was the date of surgery, when a separate biventricular circulation was established. Those without such a surgery (e.g., with only a Blalock–Taussig shunt or without any cardiac surgery) were defined as patients with palliation. Secondary pulmonary valve replacement included surgical as well as percutaneous pulmonary valve replacements.

### 2.2. Clinical Assessment

Demographic data and information on medical/surgical history were retrieved from hospital records. Symptomatic status was assessed according to the New York Heart Association classification (NYHA) as described in current guidelines [5]. Based on the results of routine transthoracic echocardiograms, left ventricular systolic function as well as right ventricular systolic function were graded qualitatively as normal, mildly-moderately, or severely impaired as described previously [6].

Coronary artery disease was diagnosed if proven by coronary angiogram or with a history of percutaneous coronary intervention or aortocoronary bypass surgery. Cyanosis was defined as resting oxygen saturation of <90%. Renal dysfunction and pulmonary hypertension were diagnosed according to clinical practice guidelines [7,8]. Lung disease included any form of it (asthma, chronic obstructive lung disease, emphysema, etc.). Patients with diabetes included both insulin-dependent and non-insulin-dependent cases.

### 2.3. Statistical Analysis

Statistical analysis was performed using SPSS version 23.0 (IBM Corp., Armonk, NY, USA) and MedCalc version 19.0.3.0 (MedCalc Software, Mariakerke, Belgium). Continuous variables are presented as mean ± standard deviation or median (interquartile range). Categorical variables are presented as numbers (percentage). A comparison between patients with TOF and PA/VSD was performed using the Student’s *t*-test for independent samples. Univariate Cox proportional-hazards analysis was used to assess the association between variables and the primary and secondary endpoints. Significant variables (*p* < 0.05) were subsequently included in a multivariable Cox proportional-hazards analysis model in a stepwise fashion. Tested variables were age at inclusion, age at correction (log), sex, TOF versus PA/VSD, liver disease, lung disease, renal disease, pulmonary hypertension, right ventricular systolic function, and NYHA for the primary endpoint with the addition of arrhythmias for the secondary endpoint.

All tests were performed two-sided and a *p*-value < 0.05 was considered significant.

This study complied with the Declaration of Helsinki, and the ethics committee of the Medical Faculty of the Technical University of Munich approved the research protocol. The requirement for informed consent was waived by the ethics committee due to the retrospective nature of the study.

## 3. Results

Altogether, 184 (41.3% female, mean age 45.3 ± 7.2 years) patients were included (Table 1). Out of these, 159 (86.4%) patients had TOF and 25 (13.6%) PA/VSD. The number of patients under follow-up increased over the course of the study period. While in 2005, thirty-one patients (27 TOF, 4 PA/VSD) over the age of 40 were under follow-up, this number increased to 93 patients (78 TOF, 15 PA/VSD) in the year 2017. Out of the 184 patients, 149 (81.0%) were in the age group 40–50 years, 22 (12.0%) in the age group 50–60 years, and 13 (7.1%) were older than 60 years.

During the patients’ lifespan at least one cardiac surgical procedure was performed in 180 (97.8%) patients, which was of corrective nature in 168 (91.3%) and of palliative nature in 12 (6.7%). One patient in TOF group and three patients in PA/VSD did not have any cardiac surgery at all.

Patients, who underwent surgical repair, underwent this in the time period between June 1958 and November 2000. The mean age at corrective repair was 10.0 ± 7.8 years.

The majority of patients had normal right (78.0%) and left (92.3%) ventricular function. Patients’ mean NYHA class was 1.87 ± 0.94.

At least one acquired comorbidity was present in 126 (68.5%) patients, while 58 (31.5%) had none. Only one comorbidity was found in 58 (31.5%), two in 33 (17.9%), and three or more in 35 (19.0%) patients. Cyanosis was present in 15 (8.2%), and pulmonary hypertension in 14 (7.6%) patients. Furthermore, 33 (17.9%) patients had systemic arterial hypertension and 11 (6.0%) hypercholesteremia, while nine (4.9%) patients were smokers. In one (0.5%) patient coronary artery disease was present and ten (5.4%) had diabetes. More detailed information is given in Table 2.

At least one interventional procedure was performed in 78 (42.4%) patients after their 40th birthday, while in 48 patients (26.1%) an electrophysiological procedure was performed after this time point.

Prior to inclusion, 55 patients (30%) had experienced arrhythmic events. These were mainly atrial arrhythmias, e.g., atrial flutter. During follow-up, 21 patients (11.4%) without prior arrhythmias experienced new-onset arrhythmic events.

### 3.1. Comparison of TOF versus PA/VSD

Patients with PA/VSD were older (*p* < 0.001) and in a higher NYHA class (*p* = 0.003) than patients with TOF. Secondary pulmonary valve replacement was also more common in PA/VSD patients (68.0%) than in TOF patients (30.8%, *p* < 0.001). Regarding comorbidities, diseases of the lung (*p* < 0.001), the liver (*p* = 0.004) as well as renal diseases (*p* = 0.005), were more prevalent in PA/VSD patients than in TOF.

### 3.2. Primary and Secondary Endpoint

After the exclusion of 15 patients who did not undergo corrective repair, 169 patients remained for Cox regression analysis (*n* = 14 PA/VSD). During a median follow-up of 3.1 years (IQR: 0.6–6.5), three (1.8%) patients died (one due to cardiogenic shock, one during an intervention, and one due to pulmonary bleeding). The primary endpoint occurred in 35 (21.0%) patients (death: *n* = 3, prevented sudden cardiac death: *n* = 10, implantation of a pacemaker after the age of 40 years: *n* = 9, new-onset heart failure: *n* = 8, new clinically significant arrhythmias: *n* = 5). On univariate analysis, older age at correction (log) [HR: 5.2, 95% CI: 1.6–16.7, *p* = 0.006], age at inclusion [HR: 1.1, 95% CI: 1.0–1.1, *p* = 0.001], right ventricular systolic function [HR: 1.5, 95% CI: 1.1–2.1, *p* = 0.018], and NYHA functional class (HR: 2.0, 95% CI: 1.3–3.1, *p* = 0.001) were predictors of the primary endpoint (MACE I) (Table 3).

**Figure 1:**
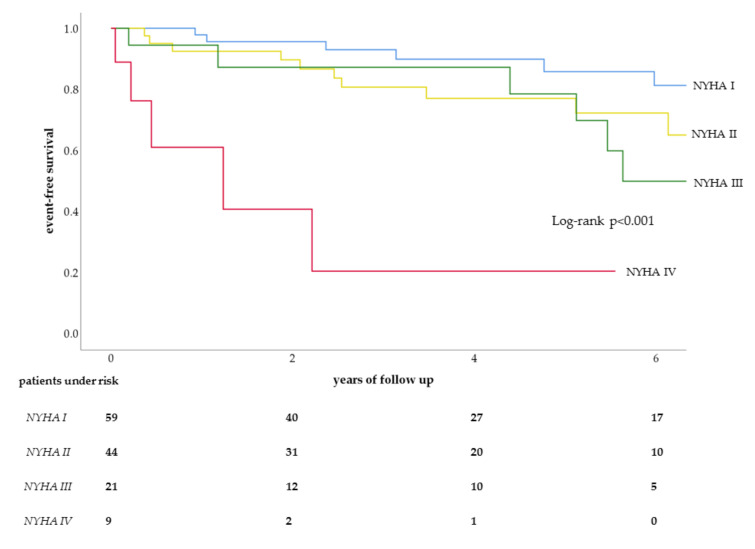
Kaplan–Meier curve for NYHA classes in MACE.

On multivariable analysis without age at inclusion, NYHA functional class [HR: 2.1, 95% CI: 1.2–3.6, *p* = 0.009] remained as an independent predictor (Figure 1). If age at inclusion was added to the multivariable analysis, both NYHA functional class [HR: 1.8, 95% CI: 1.1–3.3, *p* = 0.033] and age at inclusion [HR: 1.1, 95% CI: 1.0–1.2, *p* = 0.021] were predictors for MACE.

The secondary endpoint (MACE II) occurred in 13 (7.7%) patients during follow-up. Out of these, three were deaths and ten were prevented SCDs. At univariate analysis older age at correction (log) [HR: 13.2, 95% CI: 1.6–107.1, *p* = 0.016], age at inclusion [HR: 1.1, 95% CI: 1.0–1.2, *p* = 0.017], and prior arrhythmias [HR: 3.4, 95% CI: 1.1–10.8, *p* = 0.036] were significant predictors. On multivariable analysis without age at inclusion, only older age at correction (log) [HR: 13.2, 95% CI: 1.6–107.1, *p* = 0.016] remained as a predictor. If age at inclusion was added to the model, it remained as the only predictor of MACE II [HR: 1.1, 95% CI: 1.0–1.2, *p* = 0.014].

## 4. Discussion

Patients with TOF or PA/VSD over the age of 40 years are burdened by significant morbidity and mortality. Furthermore, a significant number of extracardiac comorbidities are often already present at this age, which were not independent predictors of outcome in our study.

Despite being one of the first CHD corrected by surgery, the life expectancy of TOF and PA/VSD patients compared to the general population is still diminished [4,9]. In our current cohort, NYHA class was predictive of a worse outcome as well as age at correction. This is in accordance with data from the large INDICATOR cohort [10]. Older age at repair was a univariable predictor of a combined endpoint composed of all-cause mortality, aborted sudden cardiac death, and sustained ventricular tachycardia [10]. In the multivariable analysis, age at repair did not remain in the final model which was mainly based of measurements from cardiovascular magnetic resonance imaging (CMR). Unfortunately, data from CMR were not available in a majority of our patients. The composition of the INDICATOR cohort was also different to ours, e.g., regarding the mean age of their patients [10]. This is also true for the cohort from the CONCOR registry (Table 4) [11]. We can expect a different population of TOF and PA/VSD patients in the future due to a corrective surgery at a younger age. Nonetheless, the number of these older patients is increasing (a nearly 3-fold increase in our center during the study period). Accordingly, a study from the UK showed a nearly 9 fold increase of ACHD patients over the age of 60 years during a 12 year period [1]. Therefore, we have to be prepared to face an aging ACHD cohort with unique challenges that might differ from those that younger ACHD patients present.

While many risk factors, including pulmonary regurgitation and RV dilatation, have been discussed for patients with TOF or PA/VSD extensively [10,12] extracardiac comorbidities have not been in the focus of attention. Especially, the significant prevalence of comorbidities in our cohort is worrisome which was higher than in the general population [13,14,15]. None of the comorbidities was an independent predictor of outcome in our study, which stands in contrast to studies in an elderly cohort with ACHD from Canada, in which multiple comorbidities were associated with worse outcomes [2]. This could be due to the much younger age in our cohort compared to the latter study (45.3 versus >65 years). In this respect, our study fills a gap, providing evidence that acquired comorbidities associated with worse outcome later in life (>60 years of age), are already present in patients within the age group of 40–60 years (93% of or cohort). But comorbidities play an important role in ACHD patients. For example, Dimopoulos and colleagues [16] studied the prevalence of renal dysfunction in 1102 ACHD patients with a variety of underlying defects and their relation to the outcome. Moderate to severe renal dysfunction was found in 9% of patients with a substantial impact on mortality. The importance of acquired comorbidities has also been shown in a study in ACHD patients over the age of 60 years [1]. Coronary artery disease was an important predictor of outcome in these patients. In our cohort, coronary artery disease was present in only one patient, probably due to the younger mean age of our cohort. Nonetheless, the prevalence of acquired cardiovascular diseases is increasing in ACHD patients as they grow as old as in the general population. This is also true for patients with TOF [9]. Therefore, systemic assessment of cardiovascular risk factors should be regularly performed, and, if necessary, preventive measures should be implemented. Unfortunately, primary and secondary prevention of acquired cardiovascular disease in ACHD is still underutilized [17].

Considering that comorbidities play such an important role and the high prevalence in our younger cohort, we are facing a perfect storm, as our patients are getting older. Therefore, preventive measures and strategies are strongly needed.

Interestingly, the endpoint of death or prevented sudden cardiac death (SCD) occurred in approximately 8% of our cohort. Prevented SCD was responsible for the majority of such events (10 out of 13). It is well known that patients with TOF or PA/VSD are prone to SCD events. In a recent study from the German National Register for Congenital Heart Defects, SCD was the cause of death in around 26% of deceased patients with TOF or PA/VSD [18]. Implantable cardioverter-defibrillators could prevent SCD—at least those with arrhythmic causes. Unfortunately, while the case and patient selection for secondary prevention of SCD are straight forward, the indication and timing for primary prevention of SCD in these patients are challenging and a matter of debate [19].

It was previously reported that an elderly cohort of ACHD patients had a high utilization of healthcare resources, which was even higher than in younger ACHD patients [1]. In our cohort, there was also a high utilization of the health care system. For example, at least one interventional procedure was performed in more than 40% of patients after their 40th birthday, while in more than 25% of patients an electrophysiological procedure was performed after this time point. Furthermore, there was a high burden of arrhythmias. At the beginning of follow-up, 30%, of patients had a history of arrhythmias (all atrial arrhythmias). During follow-up, an additional 11% of patients experienced new-onset arrhythmic events. This emphasizes the importance of previous findings. Bernier and colleagues used the Quebec database to analyze the risk of developing atrial arrhythmias in CHD [20]. The lifetime risk of developing atrial arrhythmias was 61% in patients with right-sided lesions, including TOF patients. In this regard, we have to assume that we will be faced with an increasing amount of atrial and ventricular arrhythmias in our ACHD patients as they grow older. Therefore, we need to prepare better strategies in dealing with those patients considering that ablation may be a valid option, but especially in atrial fibrillation, multiple procedures are likely to be required [21]. Early referral and careful patient selection are therefore essential to optimize results [21].

A limitation of our study is its retrospective nature with all its obvious drawbacks. In some patients, variables like the NYHA class were missing. Furthermore, cardiopulmonary exercise testing as an objective measure of exercise capacity was not regularly performed. Additionally, the cause of death was not available in all deceased patients. The number of patients with PA/VSD was rather small (13.6% of the whole cohort).

## 5. Conclusions

In conclusion, adults with TOF or PA/VSD over the age of 40 years are burdened with significant comorbidity and mortality. Preventive measures and better treatment options are needed.

## Figures and Tables

**Table 1:** Demographic data at baseline.

	All (*n* = 184)	TOF (*n* = 159)	PA/VSD (*n* = 25)	*p* *
Female	76 (41.3%)	65 (40.9%)	11 (44.0%)	0.768
Age at inclusion (years)	45.3 ± 7.2	41.0 ± 1.6	45.9 ± 7.5	<0.001
Age at corrective surgery (years)	10.0 ± 7.8	9.4 ± 7.4	16.7 ± 9.6	0.001
Pulmonary valve replacement	66 (35.9%)	49 (30.8%)	17 (68.0%)	<0.001
*NYHA functional class*				
I	60 (43.8%)	55 (45.8%)	5 (29.4%)	0.003
II	45 (32.8%)	43 (35.8%)	2 (11.8%)	
III	22 (16.1%)	15 (12.5%)	7 (41.2%)	
IV	10 (7.3%)	7 (5.8%)	3 (17.6%)	
*Rhythm*				
Sinus rhythm	129 (70.1%)	109 (68.6%)	20 (80%)	0.408
Atrial fibrillation	31 (16.9%)	27 (17.0%)	4 (12.0%)	
Paced rhythm	25 (13.6%)	23 (14.5%)	2 (8.0%)	

NYHA = New York Heart Association; *p* = statistically significant with *p* < 0.05; PA/VSD = pulmonary atresia with ventricular septal defect; TOF = tetralogy of Fallot; * TOF versus PA/VSD, statistically significant with *p* < 0.05.

**Table 2:** Prevalence of comorbidities (at baseline).

Comorbidity	All (*n* = 184)	TOF (*n* = 159)	PA/VSD (*n* = 25)
One comorbidity	55 (29.9%)	50 (31.4)	5 (20.0)
Two comorbidities	35 (19.0%)	30 (18.9%)	5 (20.0%)
Three and more comorbidities	37 (20.1%)	25 (13.5%)	12 (48.5%)
Systemic arterial hypertension	33 (17.9%)	31 (19.5%)	2 (8.0%)
Cancer	7 (3.9%)	6 (3.8%)	1 (4.0%)
Cerebrovascular events	13 (7.1%)	10 (6.3%)	3 (12.0%)
Coronary artery disease	1 (0.5%)	1 (0.6%)	0 (0.0%)
Depression	17 (9.2%)	14 (8.8%)	3 (12.0%)
Diabetes	10 (5.4%)	9 (5.6%)	1 (4.0%)
Gastrointestinal	27 (14.7%)	23 (14.5%)	4 (16.0%)
Hypercholesterolemia	11 (6.0%)	7 (4.4%)	4 (16.0%)
Liver diseases	40 (21.7%)	29 (18.2%)	11 (44.0%)
Lung diseases	30 (16.3%)	19 (11.9%)	11 (44.0%)
Pulmonary hypertension	14 (7.6%)	4 (2.5%)	10 (40.0%)
Renal disease	21 (11.4%)	14 (8.8%)	7 (28.0%)
Thyroid	41 (22.3%)	33 (20.8%)	8 (32.0%)

PA/VSD = pulmonary atresia with ventricular septal defect; TOF = tetralogy of Fallot.

**Table 3:** Univariate and multivariable predictors of primary endpoint (MACE).

Variable	Univariate		Multivariable	
	HR (95% CI)	*p*	HR (95% CI)	*p*
Age at correction (log)	5.2 (1.6–16.7)	0.006		
RV function	1.5 (1.1–2.1)	0.018		
Age at inclusion	1.1 (1.0–1.1)	0.001	1.1 (1.0–1.2)	0.021
NYHA functional class	2.0 (1.3–3.1)	0.001	1.8 (1.1–3.3)	0.033

CI = confidence interval; HR = hazard ratio; NYHA = New York Heart Association; *p* = statistically significant with *p* < 0.05; RV = right ventricular.

**Table 4:** Comparison of current study cohort to previously published cohorts from the literature.

	Current Study (*n* = 184)	Bokma et al. [11] (*n* = 575)	Valente et al. [10] (*n* = 873)	Bokma et al. [9] (*n* = 167)
Male	108 (58.7%)	326 (57%)	479 (55%)	55%
Age at inclusion (years)	45.3 ± 7.2	31 ± 11	24.4 (median)	> 50 years
Age at corrective surgery (years) median (IQR)	7.4 (5.7–11.5)	3.3 (1.4–6.7)	2.9	32% > 18 years
TOF	159 (86.4%)	534 (92.9%)	742 (85%)	n.a.
PA	25 (13.6%)	41 (7.1%)	131 (15%)	n.a.
Pulmonary valve replacement	66 (35.9%)	167 (29%)	315 (36%)	25%
Death/VT	13 (7.7%)	25 (4.3%)	32 (3.7%)	26 (15.6%)
Comorbidities	126 (68.5%)	n.a.	197 (23%)	n.a.

PA = pulmonary atresia with ventricular septal defect; TOF = tetralogy of Fallot; VT = ventricular tachycardia; n.a. = not avalable.
